# Er:YAG laser-assisted filtration surgery: initial results in rabbits

**DOI:** 10.1186/s12886-021-01986-4

**Published:** 2021-05-20

**Authors:** Noa Kapelushnik, Ari Leshno, Reut Singer, Ruth Huna-Baron, Yaniv Barkana, Alon Skaat

**Affiliations:** Sam Rothberg Glaucoma Center, Goldschleger Eye Institute, Sheba Medical Center, Tel-Hashomer, Israel, affiliated to the Sackler Faculty of Medicine, Tel-Aviv University, Tel Aviv, Israel

**Keywords:** Ab interno, Glaucoma, Minimal invasive, Trabeculectomy

## Abstract

**Background:**

Glaucoma is a leading cause of global blindness, especially preventable blindness. The increased prevalence of glaucoma has led to a growing demand for newer, safer, more rapid, and simpler treatments for the reduction of intraocular pressure (IOP). In this study, we evaluated the safety and feasibility of performing filtration glaucoma surgery with an Ab-Interno Er:YAG laser in rabbits.

**Methods:**

Nine New Zealand White rabbits age 16weeks were studied. After subconjunctival injection of mitomycin C (MMC), a novel Ab-Interno Er:YAG laser probe was inserted into the anterior chamber (AC) through a clear corneal 1mm paracentesis and directed at the trabecular meshwork adjacent to the MMC injection area. A pulsed laser beam was applied to create a patent sclerostomy connecting the AC to the subconjuctival space, resulting in a filtering bleb. The laser system used was the Er:YAG laser system - LAS25-FCU, (Pantec Biosolutions AG, Liechtenstein). Parameters used: Wave lengh: 2940nm, Pulse length: 100400sec,frequency: 250Hz. Average laser power in accordance to the fiber tip diameter: 0.85W(range 0.80.92W). Complete ocular exams, including IOP measurements, were performed on 1, 7, 14, and 23days postoperatively. Three rabbits were sacrificed on days 1, 14, and 23, and histological examinations were performed on all nine eyes.

**Results:**

All procedures resulted in a functional medium-large superior bleb without significant complications. The bleb was sustained in all rabbits by day 14 and in one of the three rabbits that reached the last follow-up at 23days. No cases of postoperative hypotony were observed. There was a transient significant reduction in mean IOP on postoperative days 5 and 7 (*P*=0.028). Histopathological analysis revealed a patent full-thickness scleral tunnel with only a minor degree of surrounding coagulation necrosis.

**Conclusions:**

The Ab-Interno laser sclerostomy procedure is potentially safe and effective based on initial experience in an in-vivo rabbit animal model.

**Supplementary Information:**

The online version contains supplementary material available at 10.1186/s12886-021-01986-4.

## Background

Glaucoma is a leading cause of global blindness, especially preventable blindness [1], with an estimate of nearly 76 million patients in 2020 and 111.8 million in 2040 [2]. Elevated intraocular pressure (IOP) is the single-most important risk factor for the development and progression of the disease [3]. The rise in IOP results from decreased flow through the primary drainage root due to obstruction of the trabecular meshwork (TM). The only currently available treatment modality is lowering IOP to a safe level which slows or halts further optic nerve damage and consequent visual field loss [4, 5]. Surgical lowering of IOP is still most commonly performed by trabeculectomy [6] following its introduction by Cairns in 1968 [7]. Reduction of IOP is achieved by creating an alternative pathway for drainage of the aqueous humor through the sclera to form a filtering bleb underneath the conjunctiva. Despite the development of newer surgical methods, the main principle remains lowering the IOP by bypassing the obstructed TM. There is a growing demand for newer, safer, more rapid, and simpler treatments for IOP reduction in light of the ever-increasing prevalence of glaucoma [2].

In this study, we report our experience with a novel delivery system of Er:YAG laser for ab-interno trabeculectomy in an animal model.

## Material and methods

### Animals

Approval for this study was obtained by the Israel national ethics committee for experiments in animals bioethics committee permission number- IL-18-10-261.

We tested the safety and efficacy of the new procedure in New Zealand White male rabbits aged 16weeks. In each rabbit one eye was randomly selected. Prior to the surgical procedure, both eyes of each test animal underwent complete ocular examination for the detection of any abnormalities. Baseline IOP was measured with a tonometer (Tono-pen XL), and color photos of both the anterior and posterior segments were taken.

### Laser system

**Fig. 1:**
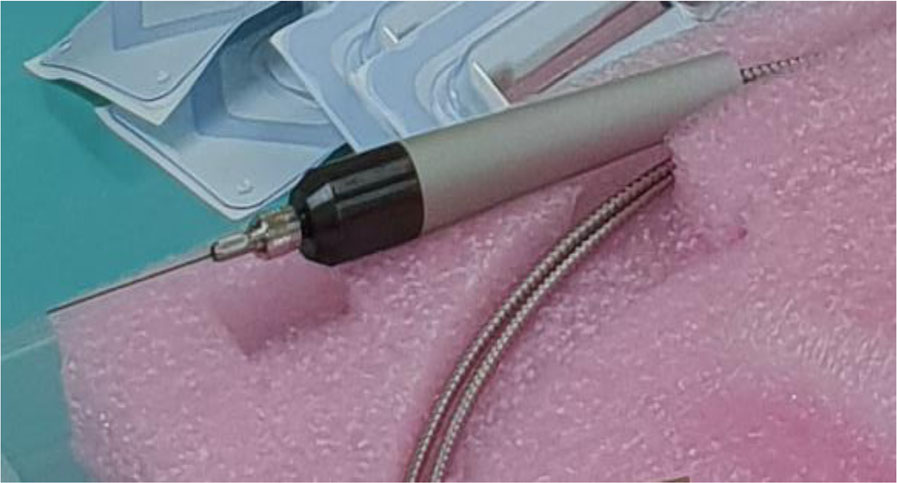
Handpiece of the thermal ablating laser system

The ab-interno procedure was performed with a thermal-ablating laser system- LAS25-FCU (Pantec Biosolutions AG, Liechtenstein). The system contains a miniaturized original equipment manufacturer laser diode driver (LDD) driving a 3mikron laser (Er:YAG) operates at a 2940nm wavelength with a pulse duration of 100400sec,a frequency of 250Hz, average output power of 0.85W (range 0.80.92W), repetition rates of up to 2kHz and delivers train of pulses. Laser energy is delivered through an optical fiber, a handpiece, and a solid core fiber-tip with a diameter of 320m (Fig.1). The fiber tip is protected against mechanical stress, and isolated from any risk of thermal dispersion by a stainless-steel tube. Further details regarding the system are available online (Additional file 1).

### Procedures

**Fig. 2:**
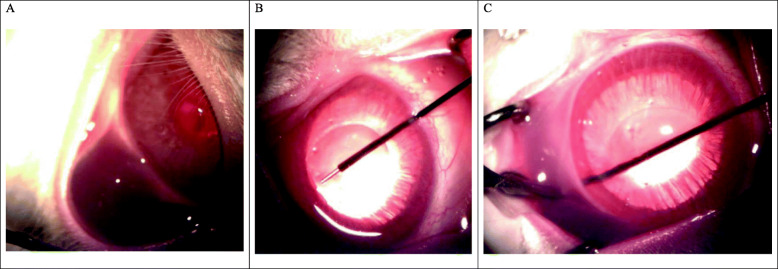
Examples of procedure stages. **a** Injection of mitomycin C into the subconjunctival space; **b** insertion of the probe into the anterior chamber through a 1mm clear corneal incision; **c** formation of the tunnel by using the ablative laser (the forceps tip points are at the end of the tip inside the formed bleb under the conjunctiva)


All procedures were performed with the animals under general anesthesia by one of the authors (AS). Mitomycin C (MMC) was injected to the superior subconjunctival space at the intended site for generating the drainage channel (Fig.2a). After filling the anterior chamber (AC) with ophthalmic viscoelastic solution (Biolon), the laser probe was inserted through an inferior 1mm clear-corneal incision (Fig. 2b). Tissue ablation was induced by activating the laser with the optical fiber tip placed juxtaposed to the target tissue, touching the trabecular meshwork, creating a new drainage channel from the AC to the subconjunctival space. Ablations were performed through a train of short pulses and high-peak power. The surgeon activated the laser ablating action and advanced the laser fiber until the fiber tip was visualized exiting the sclera into the subconjunctival space (Fig. 2c). The viscoelastic solution was re-injected into the AC to maintain AC depth at the end of the procedure. The tunnel site was marked by corneal tattooing, and Maxitrol and synthomycin 5% ointments were applied to the experimental eye. A video of the procedure can be viewed online (Additional file 2).

**Additional file 2**

### Follow-up and outcome measures

A complete ocular examination was performed on postoperative days 1, 7, 14, and 23, and it included slit-lamp examination of the anterior and posterior segments and IOP measurements with a tonometer (Tono-pen XL). All examinations were done while the animals were awake. Location, size, and functionality of the conjunctival bleb were recorded for each animal, as well as anterior chamber depth, signs of inflammation or other possible side effects (Table 1).

**Table 1:**
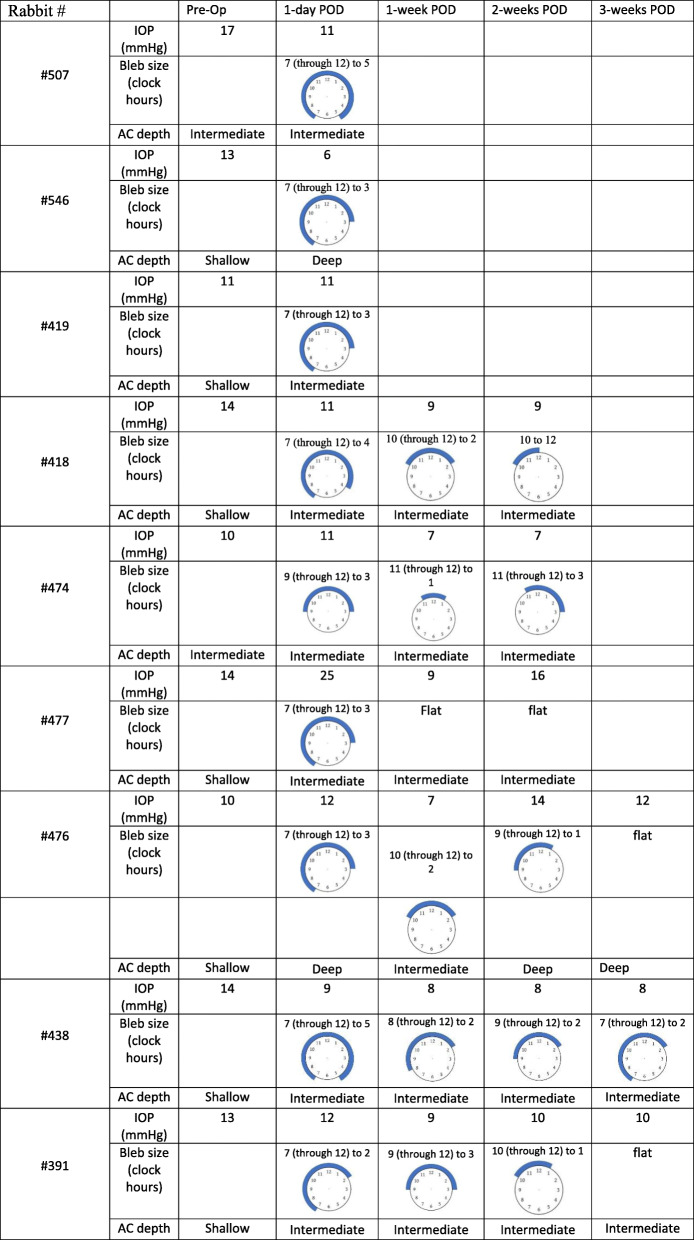
Intra ocular pressure, anterior chamber depth and bleb sizes, pre and post operatively

Three animals, each time, were euthanized on postoperative days 1, 14, and 23 by an intravenous overdose of Na-pentobarbital. The experimental eye was enucleated and preserved in formalin 4% for histological evaluations which were performed with H&E stain. The diameter and length of the ablation tunnel were measured in each experimental eye, and the samples were also evaluated for signs of collateral thermal damage.

A *P* value of <0.05 was considered statistically significant. Data were analyzed with SPSS software version 24.0 (SPSS Inc., Chicago, IL).

## Results

### Procedure

The procedure itself was uneventful, with immediate formation of a functional medium-large superior bleb in all cases. None of the experimental eyes showed signs of intraoperative corneal edema, hemorrhage, iridocorneal touch, or iris incarceration in the sclerotomy. Functional anterior blebs were observed in all cases on the first postoperative day (>3 clock hours). All eight eyes had a quiet appearance with no sign of inflammation. The AC was intermediate-deep, similar to the preoperative status, with no signs of choroidal effusion. A linear lens opacity was noted in four eyes, most likely attributable to inadvertent device contact with the anterior capsule during the procedure. The pupil was found to be dilated and non-reactive in all eight eyes, however, no iridocorneal touch or iris incarcerations were observed. One eye exhibited microhyphema, which resolved by the 7-day follow-up evaluation. Bleb formation was maintained at day 14 in five out of the six (87%) rabbits which were not sacrificed by that point. One of the three rabbits that reached the last follow-up examination on day 23 had a functional bleb.

No cases of hypotony were observed during the follow-up. There was a transient significant reduction in mean IOP on postoperative days 5 and 7 (12.92.3mmHg vs. 6.81.6mmHg and 8.20.9mmHg, respectively, paired Wilcoxon signed rank test, *P*=0.028). However, the reduction diminished and became non-significant on postoperative days 14 and 23 (10.63.6 and 10.12.0 respectively, *P*>0.05).

### Histopathological evaluation

**Fig. 3:**
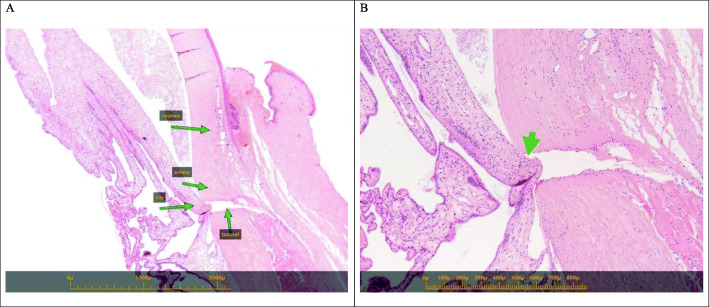
Eye 419R (One day post operation) Photomicrograph of the treated (ablated) side (**a**) with anatomical indications; **b** higher magnification demonstrating the induced tunnel and iris incarceration (arrow)

The mean ablated tunnel diameter was 133m (*n*=1), 138.990m (*n*=3), and 333.9255m (*n*=2) at the 1-, 14-, and 23-day time points, respectively. The corresponding mean ablated tunnel lengths were 560m, 410.7110.5m, and 401.2210.4m. A minor degree of coagulation necrosis was noted lateral to the tunnel formation at all three time points, but a single case at the 14days time-point,which no coagulation necrosis was detected. All examined samples (6) exhibited iris incarceration within the created tunnel (Fig.3).

## Discussion

The therapeutic application of lasers in ophthalmology and.specifically, in glaucoma is widespread and increasing. The different types of lasers allow for their utilization in a variety of conditions, such as iridotomy, trabeculoplasty, iridoplasty, and cyclophotocoagulation [8]. The rationale behind applying laser energy to create a filtering sclerostomy was that such concentrated energy can improve surgical accuracy and reduce any unnecessary tissue damage. The severity of thermal damage adjacent to the sclerostomy site is believed to be harmful to the long-term success and maintenance of fistula patency [9].The characteristics of the Erbium-doped Yttrium Aluminum Garnet laser (Er:YAG laser) make it a good candidate for the creation of long-standing intraocular openings. While the laser is capable of removing tissue by ablation [10], it is extensively absorbed by water, thus confining the energy to the desired area and tissue, with minimal thermal damage. As demonstrated in this study, the Ab-Interno Er:YAG laser system can be used safely and effectively to facilitate a connection between the AC and the subconjunctival space.

The use of Er:YAG laser for filter surgery has been explored in the past. Those attempts were met with limited success mainly due to the lack of an efficient and reliable delivery system [11, 12, 10]. There have also been several attempts to use Er:YAG laser emission in filtration surgery [11, 10, 13, 14]. The main obstacle for the application of that laser type is the lack of a reliable delivery system due to the extensive absorption in water [13].

Hill et al. [10] described their experience with Er:YAG lasers in both iridotomy and sclerostomy in pigmented rabbit eyes (Dutch Cross rabbits). Those authors used two types of laser fiber-optic probes of 600m and 400m diameters. The sclerostomies were occluded on postoperative day 1 in eight of their 10 rabbits operated by means of the 600m probe and eventually exhibited inflammation, fibrin production, and peripheral corneal decomposition. Their experience with the thinner 400m fiber optic probe was more successful. The sclerostomies remained patent until all eight animals were sacrificed in the first postoperative week, and none experienced serious complications. Using an Er:YAG laser, McHam et al. introduced a single crystal sapphire fiber optic which was found effective for delivering the laser with minimal thermal damage due to an attenuation rate compatible with the procedure and producing an effective method for an ab externo sclerotomy. Mizota et al. investigated the optimal pulse energy for sclerostomy in enucleated bovine eyes, and concluded that the optimal energy for full thickness sclerostomy with minimal thermal damage is 2mJ [15].

The procedure we followed in our study has several advantages over previously described methods. First, our delivery system used a 320m GeO2 optical fiber with a biocompatible 250m sapphire fiber tip. The small caliber enables insertion of the probe through a small (1mm) clear corneal incision such as that used in most anterior segment surgeries. In this way, this novel delivery system transformed the traditional trabeculectomy into a sutureless procedure. Furthermore, the surgical approach became more familiar to the surgeon with greater maneuverable capability and with a very short learning curve. Thanks to these advantages, the surgeon was able to position the probe at the correct position, juxtaposed to the TM, with a relative high level of accuracy, as evidenced by the adjacent thermal damage of only 55m, similar to the 50m achieved by Mizota et al. only with the assistance of an endoscopic system for direct visualization [14].

**Fig. 4:**
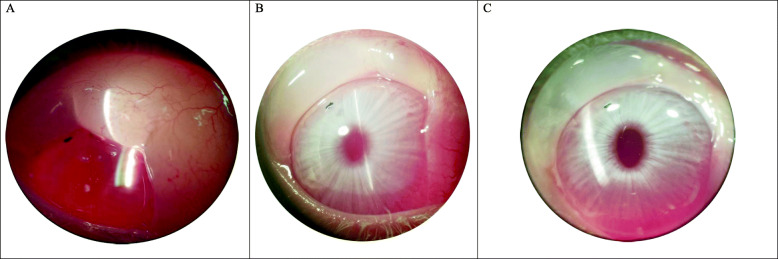
Example images of the post-operative functional bleb at day 1 (**a**) 7 (**b**) and 14 (**c**) post-op

We used MMC to reduce the possibility of occlusion of the scleral tunnel created by the Er:YAG laser. Although our sample size was small, these preliminary results indicate that the procedure is effective in creating a sustainable functional bleb (Fig.4). It should also be noted that none of the experimental eyes developed postoperative hypotony, thanks to the accurately sized scleral ablation tunnel. While we did observe a reduction in IOP, given the normal low baseline IOP, our sample size was too small to show statistical significance past the first post-operative week. Despite the use of a smaller gauge probe, we observed a high rate of iris incarceration in the histopathologic evaluation. However, since no signs of iris incarceration were observed during the in-life ophthalmological evaluation, it cannot be determined at this point whether the iris incarceration occurred during the in-life phase or post mortem during the processing phase. Regardless of the timing, it should be taken into account that the incarceration may have affected the tunnel diameter dimensions.

This study has several limitations important mentioning. Rabbits are frequently used in ophthalmology research, mainly due to the favorable size of the eye and the anatomical resemblance to the human eye, however, this model presents some limitations for our device given that the rabbit eye normally has a very narrow angle and relatively shallow anterior chamber. In addition, the structure of the iridocorneal angle and wound healing characteristics is different of that of humans. This difference can affect the fistula formation and the procedure complications when applied on human eyes. Our research histology demonstrated that despite advancement of the probe tip through the fistula into the subconjunctival space during the surgery the fistula was sized less than the probe tip size This would suggest that significant tissue shrinkage has occurred. Furthermore the dimension of the fistula at the last time point is nearly twice the sizes observed early on, with a corresponding increase in variability indicating that it might be difficult to predict the fistulas size. Albeit the small sample size, the results regarding both the intraocular pressure, functioning bleb and procedure complications were quite homogeneous, with small divergence. These results need to be confirmed in a larger study which might also help detect possible indicators for the variability in fistula size as well as determine the procedures long-term efficacy.

## Conclusion

The in-vivo application of the Ab-Interno Er:YAG laser procedure in a rabbit model is feasible and safe due to the relatively minor coagulation necrosis lateral to the tunnel formation. We confirmed that it was feasible to induce a tunnel within the sclera by means of the procedure. A current fiber diameter of 320m induced the generation of a large bleb with IOP reduction for several days in the rabbit eye, with no hypotony or flat AC. These changes in the eyes are considered to be favorable according to the recently published position paper dealing with assessment of adversity in animal research, since the intentionally induced tunnel in the sclera was well circumscribed and not associated with damage to adjacent or distant ocular structures [16]. Further studies are required to confirm the best fiber diameter for achieving the maximum efficacy and highest safety profile that will not generate any risk to human eyes.

## Supplementary Information

**Additional file 1.**

## Data Availability

The datasets used and/or analysed during the current study are available from the corresponding author on reasonable request.
