# Supplementary material for: Er:YAG laser-assisted filtration surgery: initial results in rabbits
Source: BMC Ophthalmol. 2021 May 20;21:226. doi: 10.1186/s12886-021-01986-4 (PMC8139072; doi:10.1186/s12886-021-01986-4)
Supplement: Supplementary file 2 — Additional file 1. [file 12886_2021_1986_MOESM1_ESM.docx]

**Er:YAG Laser-Assisted Filtration Surgery: Initial Results in Rabbits- Supplementary material.**

Noa Kapelushnik, Ari Leshno, Reut Singer, Ruth Huna-Baron, Yaniv Barkana, Alon Skaat

Sam Rothberg Glaucoma Center, Goldschleger Eye Institute, Sheba Medical Center, Tel-Hashomer, Israel, affiliated to the Sackler Faculty of Medicine, Tel-Aviv University, Tel Aviv, Israel

Correspondence:

Ari Leshno, MD

Sam Rothberg Glaucoma Center, Goldschleger Eye Institute, Sheba Medical Center, Tel-Hashomer, Israel

Email: arileshno@gmail.com

Tel: +972 35305828 Fax: +972 3530 2822

## Appendix 1

The system includes several components:

• LAS25-FCU – composed of the 3 mikron™ DPM-25 (Er:YAG) laser module, which is a diode pumped Er:YAG laser module with an average output power of up to 25 W and repetition rates of up to 2 kHz. The laser allows controlled and precise treatments due to the beam quality and high level of water absorption. The kit consists of the highly integrated laser module DPM-25 (Er:YAG) and the multifunctional and highly integrated laser driver LDD-20300. The laser module DPM-25 (Er:YAG) includes an integrated fiber coupling unit (FCU) for easy attachment of a ca. 250 µm core GeO2 fiber by an SMA connector.

• GeO2 fiber (ca. 320 µm core, ca. 1.5 m in length, armored, 2xSMA) including the handpiece for focusing (e.g., blepharoplasty) or the handpiece for fiber tip attachment (e.g. ab-interno laser procedure). Both the 3 mikron™ DPM-25 (Er:YAG) OEM and the GeO2 fiber were designed and built according to safety regulations for electrical laser medical device.

• Fiber tips – solid core fibers made from biocompatible sapphire material and protected by a biocompatible stainless steel tube, leaving an exposed sapphire tip of ~2 mm.

The ab interno laser procedure was performed using the Er:YAG laser system within the following operating range parameters:

Pulsed wave: working mode

Pulse length: 100-400 µsec

Frequency: range: 10-500vHz

Average laser power in accordance to the fiber tip diameter: available range 0.1-1.2 W
